# Structurally Robust and Functionally Highly Versatile—C-Type Lectin (-Related) Proteins in Snake Venoms

**DOI:** 10.3390/toxins11030136

**Published:** 2019-03-01

**Authors:** Johannes A. Eble

**Affiliations:** Institute of Physiological Chemistry and Pathobiochemistry, University of Münster, Waldeyerstr. 15, 48149 Münster, Germany; johannes.eble@uni-muenster.de; Tel.: +49-251-8355591

**Keywords:** C-type lectins, C-type lectin-related protein (CLRP), snaclecs, snake venom, hemostasis, coagulation, platelet, adhesion receptor, hematogenous metastasis

## Abstract

Snake venoms contain an astounding variety of different proteins. Among them are numerous C-type lectin family members, which are grouped into classical Ca^2+^- and sugar-binding lectins and the non-sugar-binding snake venom C-type lectin-related proteins (SV-CLRPs), also called snaclecs. Both groups share the robust C-type lectin domain (CTLD) fold but differ in a long loop, which either contributes to a sugar-binding site or is expanded into a loop-swapping heterodimerization domain between two CLRP subunits. Most C-type lectin (-related) proteins assemble in ordered supramolecular complexes with a high versatility of subunit numbers and geometric arrays. Similarly versatile is their ability to inhibit or block their target molecules as well as to agonistically stimulate or antagonistically blunt a cellular reaction triggered by their target receptor. By utilizing distinct interaction sites differentially, SV-CLRPs target a plethora of molecules, such as distinct coagulation factors and receptors of platelets and endothelial cells that are involved in hemostasis, thrombus formation, inflammation and hematogenous metastasis. Because of their robust structure and their high affinity towards their clinically relevant targets, SV-CLRPs are and will potentially be valuable prototypes to develop new diagnostic and therapeutic tools in medicine, provided that the molecular mechanisms underlying their versatility are disclosed.

## 1. Introduction

C-type lectins are a subgroup of lectins, carbohydrate-recognizing proteins, which recognize and bind carbohydrates in a Ca^2+^ ion-dependent manner [1]. They are found in all metazoan species [2,3]. Their common feature is a carbohydrate-recognizing domain (CRD), which possesses a characteristic folding pattern: the C-type lectin domain (CTLD). With the increasing number of known molecular structures of C-type lectins and of other proteins, which contain C-type lectin domains, it has become clear that they all belong to a protein superfamily, called the CTLD superfamily. However, this protein family not only comprises sugar-binding proteins, but also proteins which interact with very diverse ligands independently of carbohydrates or even Ca^2+^-ions [2,4]. Even proteins whose primary structure have very low similarities to the canonical C-type lectins belong to this CTLD superfamily, as they fold into the homologous C-type lectin fold. Among them are the link domain of the hyaluronan receptor CD44, the bacterial adhesion protein intimin and several viral proteins [5,6,7,8]. As the assignment of a protein to the CTLD superfamily is based on its structural similarity, the plethora of CTLD-containing proteins that are found in the bacterial, plant, and animal kingdoms, have numerous and very diverse ligands and functions. The vertebrate CTLD family can be subdivided into 17 subgroups, among them lecticans, collectins, selectins, the asialoglycoprotein receptor, thrombomodulin, egg-shell proteins, fish antifreeze proteins, and snake venom components [2]. The vast majority of the CTLD-containing proteins are found extracellularly, and despite their structural similarities, the members of the CTLD superfamily fulfil a large repertoire of functions.

## 2. Structure of the CTLD and its Variations with Respect to Snake Venom CTLD-Containing Proteins

The CTLD is an extremely robust folding pattern, which occurs in all metazoan species. Even parasitic bacteria and viruses seem to have taken up this protein folding motif via horizontal gene transfer [3]. The 115–130 amino acids long CTLD fold encompasses two β-sheet regions: (i) a short loop, which consists of the N-terminal and C-terminal β-strands, β1 and β5, and (ii) a three-stranded β-sheet consisting of the antiparallel strands, β2, β3, and β4. The two β-sheets are connected via a hydrophobic core, to which the highly conserved amino acid motif W–I–G–L within the β2-strand contributes. Moreover, the connection between both β-sheets is flanked on either side by two α-helices, α1 and α2, which are oriented orthogonally to each other. The three-stranded β-sheet extends into a loop between the β-strands, β2 and β3, which shows the major subgroup-specific variations. In the canonical variants, it is a long loop of about 30 residues. It may contain short stretches of α-helical or β-strand-motifs. This long loop also harbors potential binding sites for Ca^2+^ ions and for carbohydrate residues in the sugar-binding C-type lectins. Within the selectins, the long loop contains an additional pair of antiparallel β-strands [9,10,11]. The SV-CLRPs/snaclecs exclusively belong to the canonical subgroup bearing a long loop. In contrast, the compact variants bear only a very short loop between the β2 and β3 strands. Such variants are found in the hyaluronic acid-binding link domain (synonym: protein tandem repeat (PTR) domain) of lecticans, among them aggrecan, versican and other proteoglycans [12] and of the hyaluronan receptor CD44, as well as in some bacterial CTLD-containing proteins, such as in intimin and invasin [8]. Within intimin, the long loop is replaced by a single α-helix, and is even completely missing in invasin [4,6,7].

**Figure 1:**
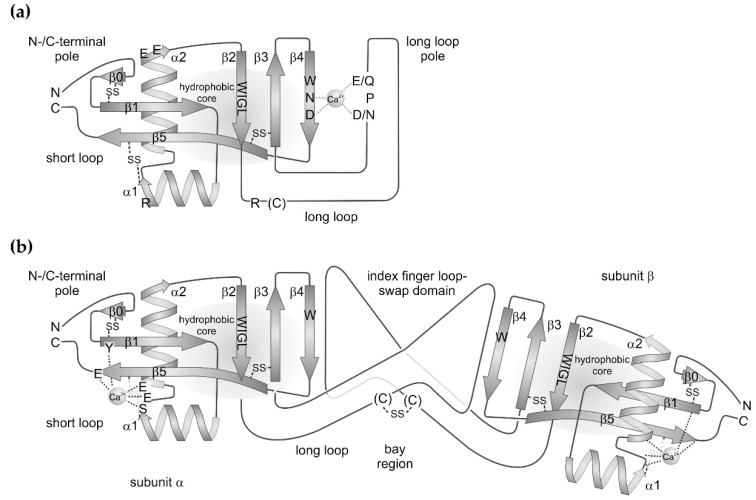
Scheme of (**a**) the molecular structures of canonical C-type lectins and of (**b**) non-carbohydrate-binding C-type lectin-related proteins (CLRPs) from snake venoms. Both protein types share a similar set of secondary structure elements. The two β-sheets, consisting of β-strands, β0–β1–β5 and β2–β3–β4. The former brings the N- and C-termini of the protein in close proximity at the N-/C-terminal pole of molecule. The latter contributes to the hydrophobic core and includes the C-type lectin-consensus sequence, W–I–G–L, within the β2 strand. The hydrophobic core is flanked by two amphipolar α-helices, α1 and α2. The highly conserved disulfide bridges are indicated. A long loop is inserted between β-strands, β2 and β3, which clearly distinguish the canonical C-type lectins from CLRPs. (**a**) In the classical C-type lectins, the long loop folds back to the β2–β3–β4 sheet, together with which it complexes a Ca^2+^ ion and thus shapes the binding site for sugar residues, preferentially galactose residues. The characteristic motifs of Ca^2+^-complexing residues are highly conserved and denoted as E/Q–P–D/N and W–N–D in the long loop and β4 strand, respectively. The W-residue of the latter is part of the hydrophobic core. Additional residues, a basic R and two acidic E residues within the helices, α1 and α2, respectively, as well as an R residue and a less conserved cysteine residue within the long loop are responsible of the assembly of this C-type lectin subunit in higher order aggregates. (**b**) In CLRPs, the long loop is expanded to an index finger loop domain, which, together with a less conserved cysteine residue, mediates the association of two different CLRP subunits into the typical CLRP heterodimers. Via this index finger loop-swap domain, the two subunits are tilted against each other along their longitudinal axis, resulting in a concave face, also called the bay region. Within the short loop, a structure-stabilizing Ca^2+^ ion complexed by glutamate, serine, and tyrosine residues of the loop connecting the two α-helices, helix α2 and β-strands, and β5 and β1. These residues are shown in subunit α and omitted in subunit β.

The short loop brings the N- and C-termini of CTLDs in close proximity and contains the two antiparallel β-strands, β1 and β5. The latter ranges from its N-terminal start in the vicinity of the β2–β3–β4 sheet to the C-terminus of the molecule (Figure 1). The short loop is largely expanded within the link domain of lecticans and CD44 with several β-strands forming another β-sheet at the N-/C-terminal pole of the CTLD [2,4]. Within the CTLD-containing components of snake venoms, the short loop is modestly expanded by one additional β-strand, β0, at the N-terminus, which aligns to the β1-strand. This N-terminal expansion is stabilized by a disulfide bridge between two cysteine residues, generically named C0 and C0′ [2]. Four additional cysteine residues, C1–C4, stabilize the protein folding and form two disulfide bridges: one between helix α2 and the C-terminal half of the β5 strand and another one between the N-terminal start of strand β3 and the loop N-terminal of strand β5. Hence, the disulfide pattern is: C0–C0′, C1–C4, and C2–C3. Additional cysteines may occur to form intra- and intercatenary bonds, thereby stabilizing a quaternary structure or a supramolecular oligomerization formed by several CTLD-containing proteins of snake venoms [2,4].

The two α-helices flank the two interconnected β-sheets, β0–β1–β5 and β2–β3–β4, on either side and are oriented orthogonally to each other. They are amphipolar helices with hydrophobic and hydrophilic residues pointing towards the protein core made of the two interconnected β-sheets and outwards towards the solvent, respectively [13].

Up to four Ca^2+^-binding sites can be found in a CTLD domain, although some of them are controversially considered as crystallographic artefacts. Moreover, not all of the four potential Ca^2+^ binding sites are simultaneously occupied in any member of the CTLD protein family. Two binding sites, sites 2 and 4, are mentioned here. Site 2 is shaped by the long loop connecting β-strands, β2 and β3, and by the β4 strand, where the characteristic amino acid sequences E/Q-P-D/N and W-N-D, respectively, participates in complexing a Ca^2+^ ion (Figure 1a). Both side chains of glutamate/glutamine and aspartate/asparagine of the long loop E/Q–P–D/N motif are brought in correct orientation for Ca^2+^ complexation by the connecting proline residue in its notable cis-configuration. In the second motif, W-N-D, the side chains of asparagine and aspartate likewise chelate the Ca^2+^ ion, while the adjacent tryptophan residue contributes to the hydrophobic core of the molecule. The Ca^2+^ ion serves as a bridge to recognize and bind the 3′- and 4′-positioned hydroxyl groups of the sugar rings. Hence, the Ca^2+^ site is characteristic of the sugar-binding C-type lectins. In contrast, the Ca^2+^-binding site 4 is not involved in sugar-binding and is located at the opposite pole of the CLTD, close to the N- and C-termini (Figure 1b). Notably among the SV-CLRP/snaclecs, this site is usually occupied by a Ca^2+^-ion [2]. It is shaped by serine and glutamate side chains located in the loop interconnecting the two α-helices and in the N-terminal portion of the α2-helix. In addition, they are supported by a glutamate residue at the C-terminal end of the β5 strand and a tyrosine side chain at the N-terminus of the β1-strand (Figure 1b) [14].

## 3. Functional Diversity of SV-CTLD

Despite their robust and well-conserved folding pattern, CTLD-containing proteins have developed an enormous functional diversity. Nowadays, they are divided in 17 groups with different functions and tissue-dependent expression [2,3]. All CTLD-containing proteins of the snake species belong to one of these 17 groups. The CTLD-containing proteins from snakes are subdivided into three subgroups. Two of them are components of the venom, whereas one subgroup of snake CTLD-containing proteins comprises snake blood proteins which associate with another protein thereby inhibiting phospholipase A_2_ [15,16]. It is responsible for inactivating phospholipase A_2_ which has pathologically reached the blood stream of the snake. As this review focusses on snake venom components, this serum CTLD-containing protein from snake blood will not be considered here further.

Although showing some variations, all snake venom CTLD-containing proteins exclusively consist of the C-type lectin domain without any additional domains. This is in contrast to other members of the CTLD family in which evolutionarily several other domains along with CTLD have been recruited into multidomain proteins to fulfil the respective functions [17,18]. The two groups of snake venom CTLD-containing proteins are (i) the sugar-binding C-type lectins and (ii) the other group which lacks sugar-binding capabilities. Because of its deficiency in carbohydrate recognition, the latter group was named snake venom C-type lectin-related proteins (SV-CLRP). Also, the name snaclecs was suggested for the latter snake venom components [19].

## 4. Sugar-Binding C-Type Lectins from Snake Venoms

**Figure 2:**
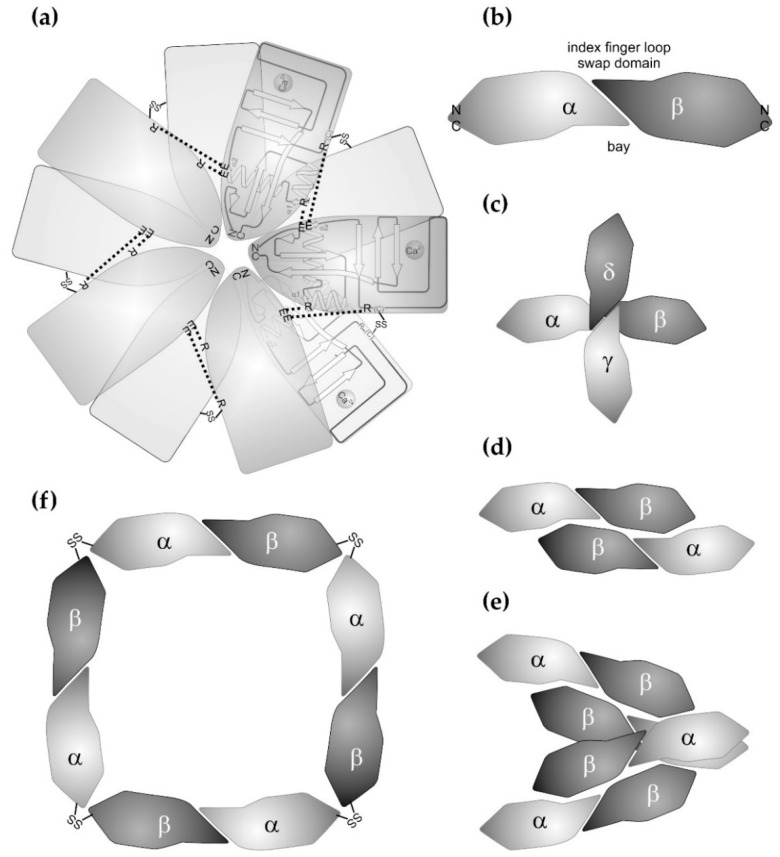
Supramolecular structures of canonical C-type lectins and C-type lectin-related proteins (CLRPs) from snake venoms. (**a**) The snake venom C-type lectins exclusively form homooligomeric structures. Ten subunits of the galactose-binding CTLD subunits from *Crotalus atrox* assemble into a double pentameric star. Each star consists of five CTLD subunits, whose N-/C-terminal pole points towards the center of the star. The pentamer is stabilized by salt bridges between glutamate and arginine residues (dashed lines). Turned around by 180° along an axis within the plain of the star, the second pentameric ring associates with the first ring and is stabilized by disulfide bridges (-SS-) between the five pairs of homodimers. The galactose-binding domains points outwards. (**b**) As a basic unit, SV-CLRPs consist of heterodimers, which dimerize via their characteristic index finger loop-swap domain in a slightly tilted manner. This results in a banana-like dumbbell shape of the heterodimeric molecule with a concave face, called the bay region. The N-/C-termini of the two subunits point in opposite directions and constitutes the two ends of the heterodimeric molecule. Such SV-CLRPs assemble into higher aggregates. (**c**) In rhodocetin, the two heterodimeric subunits form a cruciform tetrahedral molecule. The binding site for α2β1 integrin is shaped by a lateral bay region and is fully activated through conformational changes. (**d**) and (**e**) In rhodocytin/aggretin, the two heterodimers associate laterally (**d**), whereby two (αβ)_2_ aggregates even bundle up into a heterooctameric (αβ)_4_ complex (**e**). The binding sites for the CLEC-2 ligands are located at the N-/C-terminal pole of the rhodocytin α subunit. (**f**) In convulxin and flavocetin, four heterodimeric units join each other into a ring-like structure via a disulfide-stabilized head-to-tail connection at their N-/C-terminal poles. For convulxin, even a double ring assembly with a quaternary structure of (αβ)_8_ has been reported.

Most of the sugar-binding CTLD proteins from snake venoms belong to the galactose-binding C-type lectins. After the first galactose-binding lectin from snake venom was isolated from the venom of *Bothrops atrox* [20], a number of galactose-binding lectins were isolated at the protein level or identified at the cDNA level. Venom lectins known to date are especially from different *Bothrops* species (*Bothrops jararacussu*, *Bothrops pauloensis*, *Bothrops leucurus*, *Bothrops insularis*), bamboo adder (*Trimeresurus stejnegeri*), rattle snakes (*Crotalus ruber*, *Crotalus atrox*), and bushmaster snakes (*Lachesis muta*, *Lachesis stenophrys*) [21,22,23,24,25,26,27,28,29]. They were isolated by affinity chromatography with immobilized galactosyl moieties. Their abundance in venoms is in the range of 1–2% [25], but can reach 4–8% [23]. The crystal structure of the galactose-binding rattle snake lectin (RSL) from *Crotalus atrox* was the first to be resolved in 2004 [30], followed by the structure of the *Bothrops jararacussu* galactose-binding lectin (BjcuL) [31]. Based on their homologies, corresponding snake venom lectins from various other Viperidae and from Elapidae species, e.g., from *Bungarus fasciatus*, were structurally modelled [32] and revealed characteristic features. In the venom lectins of the Viperidae, but not of the Elapidae, two subunits are disulfide-linked to form a homodimer. The respective cysteine residues for this intercatenary disulfide bridge are located within the long loop, located further N-terminally than the galactose-binding site [26,30,31]. In a supramolecular array, five homodimers non-covalently associate via their lateral faces, so that two pentameric rings are formed, which are staggered against each other when viewed along the rotational axis of the pentameric stars (Figure 2a). The lateral interactions between the subunits are mediated via salt bridges between basic arginine residues and acidic side chains. The former ones are located within helix α1 and the N-terminal half of the long loop of one subunit. The latter are located within helix α2 of the neighboring subunit [30,31,32]. In addition, contacts between hydrophobic patches stabilize the supramolecular array [30,31]. Within the homodecameric star, the C-type lectin subunits are arranged in such a way that their N-/C-terminal pole point to the center of the star, and that the sugar-binding site points outwards at the tips of the rays. The Elapidae venom lectins contain an additional cysteine at their N-terminus, which was postulated to be involved in disulfide-mediated crosslinkage of two subunits into homodimers [33]. However, this putative disulfide bridge is controversially discussed as it would unlikely allow a pentameric supramolecular structure to be formed due to steric constraints [32]. Interestingly, this type of crosslinkage at the N-/C-terminal pole of CTLD is observed in SV-CLRPs, which form ring-like supramolecular arrays, but do not bind sugar residues (see below). Beyond forming such quaternary structures, snake venom lectins can form supramolecular structures of even higher order, as under galactose-deficient conditions, *Bothrops leucurus* venom lectin aggregates into fibrillar amyloids rich in β-strands, which can be visualized in electron microscopy [34].

Within the homodecameric venom C-type lectins, the sugar-binding sites are located at the ray tips of the pentameric double-star. There, a Ca^2+^ ion is complexed by the conserved motifs E/Q–P–D/N and W–N–D within the long loop and strand β4. The Ca^2+^ also complexes the two hydroxyl group of the galactose residue, mostly in position 3 and 4, and thus bridges the C-type lectin protein chain and the carbohydrate ligand [32]. Most of the published venom lectins bind D-galactosyl-residues specifically, and other monosaccharides competitively inhibit galactose binding to the venom lectin with very different selectivity and efficacy [24,35,36,37]. In 2011, the first mannose-binding C-type lectin was isolated from the venom of *Oxyuranus scutellatus* [38]. Six additional mannose-binding venom lectins from other Australian Elapidae species were reported in the same publication [38]. Another lectin from *Bungarus fasciatus* venom belongs to this group of mannose-binding venom lectins residues [33]. Noteworthy, the venom lectins show higher similarities to mannose-binding C-type lectins from plants than to the non-sugar-binding SV-CLRPs/snaclecs [32].

The functions described for snake venom lectins mostly rely on their capacity to bind to the sugar-containing glycoconjugates of glycoproteins and glycolipids, which can be inhibited by the corresponding monosaccharide in solution. One of the first observations was that galactose-binding venom lectins agglutinate erythrocyte, which has since served as an assay to determine the activity of the isolated protein and to test its selectivity for a specific monosaccharide in an inhibition test [22,25,26,36]. Whereas such erythrocyte agglutination is fatal for the envenomed victim, it fulfils the snake’s purpose to immobilize its prey or predator. However, sugar-binding proteins are employed in the innate immune system [39,40,41]. Hence, with respect to translation into medical applications, the snake venom lectins have been tested to agglutinate parasites, such as the *Leishmania* species [22]. Furthermore, by interacting with immune cells, galactose-binding lectins from the venoms of *Bothrops jararacussu* and *Bothrops leucurus* stimulate peripheral mononuclear cells and neutrophil granulocytes, respectively, to produce more reactive oxygen species, a characteristic sign of the respiratory burst during inflammation [42,43]. In addition, increased vascular permeability and edema formation, as well as leukocyte infiltration along post-capillary venules have been described for the galactose-binding *Bothrops jararacussu* lectin (BjcuL) [42]. In contrast, the closely related *Bothrops pauloensis* lectin (BpLec) did not induce such inflammatory responses in vivo, but showed opposing effects on angiogenic sprouting in in vitro vs. in vivo assays [44]. The most impressive effect that will potentially be exploited in medical applications is that the galactose-dependent *Bothrops jararacussu* lectin (BjcuL) very efficiently disrupts biofilm formation of microbiological pathogens [31,45]. This feature might be of use to avoid the biofilm-assisted pathogen colonization of medical implants or endoprosthetic devices.

## 5. Snake Venom-C-Type Lectin-Related Protein (SV-CLRPs)/Snaclecs

The other type of CTLD-containing proteins of snake venoms are SV-CLRPs. Until a few years ago, they had been described only for venoms of Viperidae [46]. Although being most abundant in the Viperidae venoms, recent discoveries of CLRPs in the venoms of Elapidae and Dipsadidae snakes (formerly called Colubridae) suggest that the protein families of C-type lectins and non-sugar-binding CLRPs separated before the snake family diverged, and that the SV-CLRPs majorly evolved in the Viperidae species with an impressive divergence [47]. In contrast to the venom lectins, the basic structure of an SV-CLRP is a heterodimer, consisting of two highly homologous CTLD subunits, α and β (Figure 1 and Figure 2). Both subunits, α and β, show low primary sequence similarities to the sugar-binding venom lectins of about 14–37% and 25–35%, respectively [32].

The molecular structure of the SV-CLRPs is similar to C-type lectins (Figure 1b). The short loop contains an N-terminal extension, which usually folds into a β-strand. This β0 strand aligns with the β1 strand, and an additional disulfide bridge connects both strands. The two α-helices flank the molecule, and the second β-sheet is formed by the strands β2, β3, and β4 similarly to the C-type lectins. However, the snake venom lectins and CLRPs/snaclecs most strikingly differ in their long loop. The residues E/Q–P–D/N constituting the Ca^2+^-dependent sugar-binding site are missing entirely (Figure 1b). The β4 strand contains only a rudimentary motif W–x–N, with x being a large hydrophobic residue or a hydrophilic serine/threonine residue. Therefore, the latter motif is unable to assist complexation of a Ca^2+^ ion [32,48].

Within the SV-CLRPs heterodimers, the two subunits join frontally with their long-loop poles and hence are completely different from the venom lectin homodimers. The long loop mediates the firm heterodimerization of the two subunits. In contrast to the venom lectins, it is expanded and points away from the CTLD core like an index finger and is stabilized by the so-called domain swapping. The index fingers of the two CTLD subunits, α and β, align in an antiparallel and slightly twisted manner, thus appearing as they would hook up with each other (Figure 1b). Thus, the index finger loop of one subunit reaches far to the core region of the other subunit, providing a large contact face and allowing conformational changes to be transmitted mutually between the two subunits. Some, but not all, SV-CLRPs/snaclecs bear a cysteine residue within the long loop at a position different from the one of the venom C-type lectins. It crosslinks the two subunits of the heterodimeric SV-CLRP via an intercatenary disulfide bridge between the two index finger loops. Moreover, as the axes of the index fingers are slightly tilted against the axis of the subunit core, domain swapping results in a banana-like dumbbell shape of the CLRP heterodimer with a bay region formed by the slimmer domain-swapping region and flanked by the two bulkier core domains of either subunit (Figure 1b and Figure 2b). It is this concave face of the CLRP heterodimer, which was proposed to be the general binding region for the CLRP ligands. However, the CLRP heterodimers proved to be extremely versatile and it seems that multiple interactions with ligands are possible with almost any face of the banana- shaped SV-CLRP heterodimeric molecule.

Related to the different interaction sites, SV-CLRPS/snaclecs show a high versatility of oligomerizing into aggregates of higher supramolecular order (Figure 2). Various quaternary structures have been described: αβ, (αβ)_2_, (αβ)_3_, (αβ)_4_, and (αβ)_8_ as well as αβγδ, or αβα’β’, whereby the latter are two pairs of highly homologous subunit heterodimers [49,50,51,52,53,54]. Interacting with each other via their lateral face or their joint N-/C-terminal poles, the SV-CLRPs assemble into tetrahedral, bundle-like, and ring-like supramolecular structures (Figure 2c–f). Also, a C3-symmetric bundle of three tilted heterodimers is deposited in the protein data base [14]. Being multivalent, they also present binding sites for various ligands with a characteristic topography and orientation, and may induce clustering of their ligands. If the ligands are cellular receptors, the cells may be forced by multivalent CLRPs to cluster their receptors in a specific manner. In addition to ligand occupancy, this topography-dependent presentation of binding sites may be a relevant parameter, which determines cellular signalling in an agonistic or antagonistic manner [55,56,57]. The formation of higher aggregates with different symmetries (tetrahedral, bundle-like, ring-like, and double ring-like structures) is supported by cysteine residues which are found at the N- and C-terminal ends of some SV-CLRPs, e.g., flavocetin and convulxin [50,54,58,59]. They allow a covalent crosslinkage of two heterodimers via their N-/C-terminal pole in a head-to-tail connection.

SV-CLRPs/snaclecs are highly promiscuous in their ligand spectrum [48,60,61,62]. They target clotting factors and various receptors on platelets, endothelial cells, and immune cells. Some of these interactions are inhibitory and interfere with the interactions of the target molecules with their endogenous ligands. At the cellular level, some interactions of SV-CLRPs with their target receptor molecules act antagonistically on cells. Conversely, some other CTLD-containing venom proteins, despite their similar molecular structure, stimulate the respective cellular functions as agonists. These opposing effects may be partially due to the aggregation number and geometry of the supramolecular SV-CLRP aggregates. This may explain the different effects of SV-CLRPs directed towards surface receptors for platelets, as receptor clustering is the key determinant in signalling and hence activation of platelets [54,55,56]. This yet unpredictable feature of being either agonist or antagonist, and the fact that CLRPs show high promiscuity in their selectivity of target molecules, makes it extremely difficult to subgroup the numerous SV-CLRPs/snaclecs according to their functions [17,48,63]. Therefore, irrespective of their activating or inhibiting biochemical potential, irrespective of their agonistic or antagonistic effects on cells, and irrespectively of the different target molecules, the SV-CLRPs/snaclecs will be grouped in the following paragraphs according to the physiological system which they affect. They play a crucial role in hemostasis, a complex system to stop bleeding, which encompasses coagulation, platelet activation, and thrombus formation [64,65,66,67]. Moreover, they affect endothelial cells, which line the blood vessels, act as a barrier between the blood stream and tissue, mediate exchange of nutrients, and regulate diapedesis of immune cells during inflammation [18,68,69,70]. Despite being grouped according to these effects on coagulation, on platelet aggregation, and on endothelial cells in the following paragraphs, the same CTLP-containing snake venom component may be named more than once due to its promiscuous binding pattern and due to the overlapping targeting spectrum of some SV-CLRPs.

### 5.1. SV-CLRPs Targeting Clotting Factors

To prevent blood loss, the closed circulatory system of a vertebrate has a self-sealing system, activated during vessel and tissue injury, which is temporarily and spatially restricted to the injury site, and which promotes the speedy healing of the tissue after damage [64,65]. Fibrinogen, an abundant blood serum protein, plays a crucial role in hemostasis. Upon proteolytic cleavage by thrombin, it is converted from a soluble blood component to an insoluble fibrin molecule. Fibrin molecules aggregate into highly ordered bundles, which seal the wound by withstanding tensile forces and serve as a preliminary extracellular matrix for the regeneration of tissue [71]. The conversion of fibrinogen to fibrin is a complex and fine-tuned process orchestrated by a system of coagulation factors, which in a cascade-like process subsequently activate each other. The immediate activator of fibrinogen is thrombin, a coagulation factor, which itself is activated by factor X in complex with factor V [65]. Factor X can be activated by two routes, the intrinsic and extrinsic pathway, whereby a factor IX–factor VIII-complex and factor VII, respectively, proteolytically act on factor X. The activity of the interdependent coagulation factors is checked and balanced by additional regulatory proteins. As part of this regulatory network, thrombin and factors VII, IX, and X, are post-translationally γ-carboxylated at specific glutamate residues within certain domains, the so-called γ-carboxyl-glutamate (Gla)-domains. Via these Gla-domains, these factors complex Ca^2+^ ions and are thus tethered to lipids of the platelet membrane, a necessary step of coagulation. Being such a delicately balanced system of key physiological importance, the coagulation cascade and fibrin conversion are targets of different snake venom components. Among them are SV-CLRPs, which can act as coagulants or anticoagulants, resulting in disseminated intravascular coagulopathies (DIC) or severe bleedings, both life-threatening situations [18,66,67].

As most coagulation factors are serine proteinases, the coagulation cascade is disturbed especially by exogenous serine proteinases and metalloproteinases, which are abundant components of snake venoms [17,72,73,74,75]. By inappropriately cleaving the endogenous clotting factor, the snake venom serine proteinases (SVSPs) or snake venom metalloproteinases (SVMPs) can activate the coagulation cascade and result in fibrinogen conversion. Although the SV-CLRPs do not play a prominent role as coagulants, it is interesting that the factor X activator from Russell’s viper (*Daboia russelii*), RVV-X, is a snake venom metalloproteinase which is disulfide linked to a CLRP heterodimer [76]. A C-terminal cysteine residue of the CLRP subunit α connects the N-/C-terminal pole of the CLRP heterodimer to the C-terminal domain of the metalloproteinase domain [76]. With its CLRP moiety, this venom factor X activator binds to the Gla-containing domain of the endogenous blood coagulation factor X. With it metalloproteinase domain it cleaves and thus activates the endogenous factor X, resulting in an uncontrolled activation of thrombin and consequently in an inappropriate conversion of fibrinogen to fibrin. Although few examples are known to date, activators of factor X and factor V from other snake venoms, such as the factor V-activating carinactivase-1 from *Echis carinatus*, may share this molecular structure and mechanism involving a CLRP moiety [62,77].

SV-CLRPs that inhibit coagulation factors are rather rare or undiscovered, but botrojaracin from *Bothrops jararaca* binds with high affinity to thrombin, thereby allosterically blocking its fibrinogen-converting activity [78,79]. Moreover, it also binds to prothrombin and blocks its proteolytic activation [80]. More commonly found are the anticoagulant SV-CLRPs, which bind to the Gla-domains of factor X, factor IX, or both of them, thereby inhibiting the respective coagulation factor [14,81,82,83,84,85]. The Gla-containing domain of the coagulation factors is recognized by the bay region of the SV-CLRPs. Only one direct contact of the CLRP exists between a glutamate side chain of subunit α and one of the numerous Ca^2+^ ions complexed by the γ-carboxyl-glutamate residues of factor IX. Most contacts within the large contact interface between CLRP and the Gla-domain of factor IX are mediated via direct protein–protein interactions, especially via hydrophobic patches. Nevertheless, the interaction between both partners requires two Ca^2+^ ions to be complexed by either of the two CLRP subunits within their short loop region, distant from the ligand binding interface [83]. These Ca^2+^ ions are located between helix α1 and the C-terminal end of β5 strand and are complexed by serine, glutamate, and glutamine side chains close to the N-/C-terminal pole of the CLRP molecule [83,84]. Removal of Ca^2+^ ions induces a conformational change within the factor IX-binding CLRP of *Trimeresurus/Protobothrops flavoviridis* [86].

The direct protein-protein interaction between the factor X- and factor IX-binding proteins with the Gla-domain may explain the preference of some SV-CLRPs for one or the other coagulation factor. Halyxin from *Agkistrodon/Gloydius halys brevicaudus* binds to both FIX and FX, but not to other Gla-containing coagulation factors [87]. Factor X-binding protein (FX-bp) from *Agkistrodon acutus* binds with higher affinity to factor X than to factor IX, with the dissociation constants differing by a factor of about 7 [88,89]. In contrast, factor IX-binding protein (FIX-bp) from *Echis carinatus leucogaster* clearly prefers binding to factor IX over factor X, with a ratio of dissociation constants even approaching a value of 20 [81]. A similar preference occurs with FIX-bp isolated from *Trimeresurus flavoviridis* [82], while the homologous protein from *Agkistrodon halys pallas* venom exclusively binds to FIX [90].

The potential of these anticoagulant SV-CLRPs has not really been harnessed pharmacologically yet, although their strong inhibitory masking of the Gla-domains of hemostatically essential clotting factors might be a clearly defined molecular target for the development of new anticoagulants. In contrast, the SV-CLRP-containing factor X and factor V activating proteases, RVV-X and carinactivase-1, have been used as diagnostic tools to measure the coagulation status of patients [49,91,92,93]. Due to the CLRP-mediated specificity to Gla-residues, factor V activating carinactivase-1 is used to selectively quantify the Ca^2+^-activatable prothrombin levels in the blood of patients under vitamin K-dependent anticoagulant therapy [94,95,96]. Moreover, the use of RVV-X helps distinguish bleeding disorders caused by factor X deficiency from coagulopathies based on deficiencies of other coagulation factors [97].

### 5.2. SV-CLRPs Targeting Platelet Receptors

Platelets are easily accessible and tangible targets for snake venoms [19,60,61,70,98,99]. They express receptors on their surface, which bind to extracellular matrix molecules, such as von Willebrand factor (vWF), collagen, and fibrin, which becomes accessible or available during vessel damage and coagulation, respectively [71,99,100,101,102]. vWF and collagen especially provide signals to the platelets. As a consequence, activated platelet rearrange their cytoskeleton and take a dendritic shape, degranulate and release additional signal molecules. Moreover, they activate adhesion receptors, such as the fibrin-binding platelet receptor αIIbβ3, which eventually mediates firm attachment to the fibrin clot and its contraction [64,65,103]. The initial steps of vWF- and collagen-induced platelet activation is mediated by the vWF-binding glycoprotein (GP) Ib–V–IX-complex and by the two collagen-recognizing receptors, GPVI and integrin α2β1 [98,104]. Ligand occupancy and clustering of these receptors play an essential role in triggering platelet activation [55,56].

**Figure 3:**
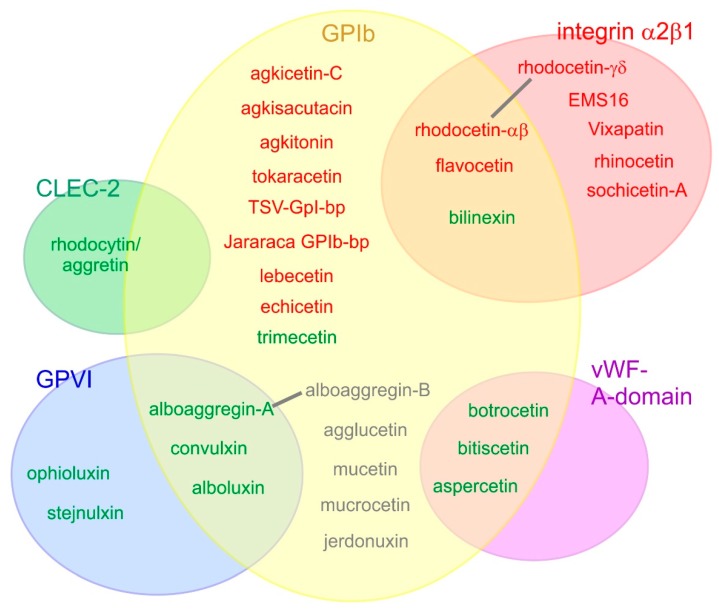
Platelet receptors are targeted by various snake venom CLRPs. Five different platelet receptors have so far been identified as targets of SV-CLRP: the glycoprotein (GP) Ib, integrin α2β1, von Willebrand factor (vWF) A-domain (which interacts with GPIb), as well as GPVI and CLEC-2. SV-CLRPs that activate platelets and agonistically cause their aggregation are indicated in green; those ones which only aggluinate platelets are shown in gray; and inhibitory and antagonistically platelet-blocking SV-CLRPs are indicated in red. Overlapping receptor specificities were observed for several SV-CLRPs. For some of them, such as rhodocetin and alboaggregin, the binding sites for different receptors are located on different heterodimeric subunits. For others, the mechanism of recognizing different receptors has remained elusive. Depending on the binding partner, SV-CLRPs employ different interaction sites; e.g., on one hand, their concave face for binding of integrin α2β1 and vWF-A-domain (rhodocetin-γδ, EMS16, botrocetin, bitiscetin, albeit with different orientation with respect to the receptor), on the other hand, their N-/C-terminal pole for CLEC-2 binding (rhodocytin/aggretin).

SV-CLRPs show a broad and partially overlapping platelet receptor-binding spectrum (Figure 3). [19,48,60,104]. All of them are active in a non-enzymatic manner. Some of them trigger a signal and induce platelet activation, resulting in inadequate thrombus formation and vessel occlusion. Others inhibit binding of the physiological ligand and thus antagonistically prevent the receptor from eliciting a signal, resulting in severe bleeding. Moreover, several SV-CLRP recognize different platelet receptors, affecting several signalling pathways within platelets.

Von Willebrand factor (vWF) is a multidomain protein and binds via its A3 domain to collagen and via its A1 domain to the vWF-receptor, a complex of the membrane proteins GPIb α, GPIb β, GPIX, and GPV in a stoichiometry of 2:2:2:1 [105,106]. The N-terminal domain of GPIBα is a leucine-rich domain that has a horse shoe-like curvature. Its concave face is the binding site of the vWF–A1 domain. Several SV-CLRPs were identified to inhibit GPIb and antagonistically block vWF-induced platelet aggregation (Figure 3). Among them are agkisacutacin, agkicetin C, and akitonin, all three from *Agkistridon acutus* [107,108,109,110,111], flavicetin, tokaracetin, and TSV-GPIb-pb from *Trimeresurus flavoviridis*, *Trimeresurus/Protobothrops tokarensis*, and *Trimeresurus stejnegeri*, respectively [112,113,114], jararaca GPIb-bp from *Bothrops jararaca* [112,115], lebecetin from *Macrovipera lebetina* [116], echicetin from *Echis carinatus* [117,118,119], and rhodocetin subunit αβ from *Calloselasma rhodostoma* [120]. In contrast, some SV-CLRPs were reported to agglutinate platelets via binding to GPIb: agglucetin from *Agkistrodon acutus* [51,52], alboaggregin-B from *Trimeresurus albolabris* [121,122,123], mucrocetin and mucetin from *Trimeresurus mucrosquamatus* [53,124], as well as jerdonuxin from *Trimeresurus/Protobothrops jerdonii* [125]. For jerdonuxin and mucetin, an increased signalling in platelets was detected on the basis of tyrosine phosphorylation of signalling proteins in response to the GPIb-binding SV-CLRP [124,125]. Mucrocetin induces aggregation of platelets in a GPIb-dependent manner, but it is not clear yet whether the platelets agglutinate due to the oligomeric nature of mucrocetin or whether the CLRP agonistically induces signalling and activation of platelets [53]. Agglucetin and alboaggregin-B bind to the vWF-receptor but do not induce an increase of intracellular Ca^2+^ ions and do not trigger degranulation, respectively, both signs of platelet signalling [51,52,122]. This suggests that the platelets can be crosslinked and agglutinated by SV-CLRP-mediated GPIb multimerization, whereas a physiological agonist elicits an active signalling process.

The interaction of these GPIb-binding SV-CLRPs has not been studied at the molecular level yet, due to the lack of crystallized complexes. In one instance, alboaggregin-B was shown to inhibit vWF from binding to GPIb, indicating that this SV-CLRP likely bind to a site within GPIb which is identical or overlapping with the vWF-binding site [123].

In contrast, there are SV-CLRPs which bind to a complex of GPIb and vWF (Figure 3), among them botrocetin from *Bothrops jararaca*, bitiscetin from *Bitis arietans*, and aspercetin from *Bothrops asper* [126,127,128,129]. The molecular structure of the trimeric complexes encompassing GPIbα, vWF–A1 domain, and botrocetin has been solved [126]. Although both CLRP heterodimers bind to the vWF–A1 domain with their concave face, they bind with distinct orientations, and perpendicularly to each other, to the globular vWF–A1 domain [63,126,127,128,130]. Again, this underlines the versatility of CLRPs. With respect to their interaction with GPIb, both botrocetin and bitiscitin do not induce any conformational change within the vWF-receptor. Moreover, neither the vWF–A1 domain nor the CLRP undergoes a conformational change [131]. Hence, their biological activity to induce platelet activation in the presence of blood plasma vWF is likely caused by stabilization of the vWF–GPIb-binding by acting like a molecular brace [126,127,128]. This might be physiologically relevant, as vWF-induced platelet activation occurs under high shear forces at maximum flow rates in the arterial vessel system [98,99,101,105].

Integrin α2β1 is a collagen-binding member of the large family of cell adhesion molecules consisting of two subunits, α and β [132,133,134,135]. Both integrin subunits are anchored via a transmembrane domain within the cell membrane. The extracellular domains of both subunits form one head domain, which harbours the binding site for the extracellular matrix ligands [136]. Upon ligand binding, integrins undergo major conformational changes between a bent and an upright/activated conformation, which is transduced to the intracellular domain [134,137]. Lacking any kinase domain, the cytoplasmic domains of integrin recruit cytoskeletal proteins, adaptor and signalling proteins. This enables not only firm connection between the cytoskeleton and the extracellular matrix, but also signal transduction via the integrin-mediated cell-extra cellular matrix contacts [137,138]. Moreover, integrin signalling also encompasses clustering of several integrin molecules and associated proteins into specific cell-adhesive cell organelles, termed adhesomes [139]. Collagen-binding integrins are a subgroup within the 24-membered integrin family. They characteristically bear an additional A-domain, which is inserted into their head domain. This insertion- or A-domain is homologous to the vWF–A domain [135]. Within the subgroup of collagen-binding integrins, integrin α2β1 is unique, inasmuch as it is the only integrin that is not targeted by the large family of snake venom disintegrins but selectively by SV-CLRPs [140,141]. The first SV-CLRP which was identified to target integrin α2β1 was rhodocetin from *Calloselasma rhodostoma* [142,143]. This discovery was followed by the identification of EMS16 from *Echis multisquamatus* [144], lebecetin from *Macrovipera lebetina* [145], vixapatin from *Vipera lebetina* [146], rhinocetin from *Bitis gabonica rhinoceros* [147], and sochicetin-A and B from *Echis sochureki* [148] (Figure 3). In contrast to the first reports [142,143], rhodocetin was identified as a heterotetrameric SV-CLRP consisting of four CTLD subunits, α,β,γ, and δ [149]. The two pairs of subunits, αβ and γδ, are firmly associated via the index finger loop-swap domain. Remarkably, upon binding to integrin α2β1, the subunits αβ and γδ, fall apart [150]. While the released rhodocetin-αβ binds to GPIb and neuropilin-1 on the platelets and endothelial cells, respectively [120,151], rhodocetin-γδ stays firmly attached to the A-domain of integrin α2β1 [152]. Rhodocetin-γδ binds in a different orientation to the integrin α2 A-domain than botrocetin and bitiscetin bind to the vWF-A domain, although the integrin A-domain and the vWF–A1 domain share a similar structure [126,127,128,152]. Also opposed to botrocetin and bitiscetin, rhodocetin-γδ induces a conformational shift within the integrin α2 A-domain and brings it to an inactive conformation. Thus, it shuts off integrin α2β1 signalling in addition to its steric blockage of the collagen-binding crevice on top of the A-domain [152]. Also unprecedentedly, upon integrin α2β1 binding, rhodocetin itself undergoes a conformational change not only in its quaternary structure but also within the rhodocetin-γδ. This molecular mechanism of mutually induced conformational changes explains the firm binding of both partners [152]. Specificity towards α2β1 integrin is achieved by a tryptophan residue of the rhodocetin-γ subunit which perfectly stacks above two glycine residues. These two glycine residues shape a shallow dimple at the lateral face of the integrin α2 A-domain. They are preserved among the integrin α2 subunit of the various vertebrate species, but unique to the integrin α2 sequence within the integrin family [152].

It is noteworthy that some SV-CLRPs, such as rhodocetin from *Calloselasma rhodostoma*, flavocetin from *Trimeresurus flavoviridis*, and bilinexin from *Agkistrodon bilineatus*, can bifunctionally block two platelet receptors, integrin α2β1 and GPIb [120,150,153,154]. Whereas flavocetin can bind to both receptors simultaneously [153], the tetrameric rhodocetin harbours the binding sites for the two receptors on the two heterodimeric subunits, αβ and γδ, which dissociate as part of the binding mechanism [152].

Integrin α2β1 is the only collagen-binding integrin on platelets, but not the only collagen receptor on platelets [98,103]. In addition to integrin α2β1, the glycoprotein (GP) VI is another receptor for collagen on platelets [155,156,157]. In a non-redundant, but complementary manner, both receptors transduce the strongly stimulating signal of collagen into platelets by partially independent pathways [55,156,158]. They also have different binding prerequisites towards collagen. Moreover, integrin α2β1 was hypothesized to be responsible for strong adhesion of platelets to collagen, necessary for the formation of a shear stress-resistant thrombus [159]. GPVI belongs to the family of immunoglobulin-fold containing receptors and signals in its dimerized form via associated signalling molecules, such as the Fcγ-receptor [157]. It is targeted by several SV-CLRPs (Figure 3), such as ophioluxin from *Ophiophagus hannah* [160], stejnulxin from *Trimeresurus stejnegeri* [161], convulxin from *Crotalus durissus terrificus* [162,163], alboluxin from *Cryptelytrops/Trimeresurus albolabris* [164], and alboaggregin-A from *Trimeresurus albolabris* [122,165,166]. The latter three were reported to bind also to GPIb (Figure 3). Convulxin is the best-studied example of this group of GPVI-binding SV-CLRPs. Convulxin competes with GPVI-binding competent synthetic collagen-related peptide, which specifically are hydroxyproline-free and bundled, for the binding site within GPVI [167]. Three tyrosine residues within the GPVI were mapped to be part of the convulxin binding site of the receptor [168]. The signalling pathway underlying the agonistic activation of platelets by convulxin was also disclosed [157,163,169,170]. It is noteworthy, that convulxin occurs not only in an annular quaternary structure of four heterodimeric subunits [58,59], but also as a double ring with the molecular formula (αβ)_8_ [54]. This supports the hypothesis that not only is there high affinity binding of the SV-CLRPs to their receptor ligands, but also their multivalency clusters several platelet receptors, thus strongly reinforcing their agonistic action [56,57].

C-type lectin-like receptor 2 (CLEC-2) is a type II transmembrane receptor with a short N-terminal cytoplasmic tail containing the amino acid sequence Y–x–x–L which encompasses a singular half of the usual, in tandem occurring immunoreceptor tyrosine activation motif (ITAM), hence called hemiITAM motif. Moreover, CLEC-2 consists of a transmembrane domain, a juxtamembrane neck domain, followed by the C-terminal CTLD domain. The latter binds neither Ca^2+^ ions nor carbohydrates. It is expressed on a subset of immune cells, such as dendritic cells, monocytes and neutrophils, and abundantly on megakaryocytes and platelets [171,172]. Before the endogenous ligand podoplanin was known, rhodocytin/aggretin from the venom of the Malayan pit viper (*Calloselasma rhodostoma*) was identified to be the CLEC-2-targeting SV-CLRP [173,174,175] (Figure 3). In fact, the CLEC-2-initiated signalling cascade resulting in platelet activation and aggregation was not elucidated with podoplanin, but with rhodocytin/aggretin. It has a tetrameric quaternary structure, (αβ)_2_, as two αβ heterodimers bundle up laterally in the crystal structure [176,177]. By this lateral association into ordered oligomers of even higher aggregation numbers, rhodocytin causes dimerization of at least two CLEC-2 molecules on the platelet surface. Thus two hemiITAM motifs comes in close vicinity and complement into an ITAM motif [178,179]. Consequently, CLEC-2 homodimers are recruited into lipid rafts, a prerequisite for subsequent CLEC-2 phosphorylation/activation by the signalling molecule Syk [179,180,181,182]. Syk is a crucial molecule in platelets which also signals downstream of other platelet receptors, such as the collagen receptor GPVI and the platelet integrin αIIbβ3. Together with Syk, phosphorylated CLEC-2 homodimers recruit Src family members, which activate downstream effector proteins, eventually resulting in platelet activation and thrombus formation [157,183,184].

The CTLD domain of CLEC-2 shows a robust structure and possesses a positively charged patch of four arginine residue on its lateral face [185,186]. They serve as contact site for both ligands, rhodocytin and podoplanin. Within an E–D–x–x–x–T motif of podoplanin, two acidic side chains of adjacent glutamate and aspartate residues and a sialic acid residue of the threonine-anchored O-glycan form salt bridges with the arginine residues of CLEC-2. Mimicking this binding pattern partially, the SV-CLRP rhodocytin uses glutamate and aspartate residues within its N-terminal E–D–x–D motif, as well as the C-terminal tyrosine residue to form an interaction face that is complementary to the arginine residue patch of CLEC-2 [185]. Again showing the flexibility of SV-CLRP, rhodocytin uses its N-/C-terminal pole as a contact face to its target molecule.

In addition to its role in the development of the lymphatic vessel system [187,188], CLEC-2 has attracted major attention in tumour biology in recent years. Several tumour entities express podoplanin. Upon their metastatic dissemination into the blood stream, they interact with platelet inter alia via the podoplanin–CLEC-2 axis [175,189,190,191]. Thereby, blood-borne tumour cells recruit platelets, which cover the tumour cells and thus protect them from immune attack, support them with necessary growth factors, and provide their adhesive capability to the tumour cells for extravasation and metastasis [192]. As platelets are an indispensable partner for haematogenous metastasis [193], blockage of CLEC-2 with rhodocytin prevents platelets from being recruited to tumour cells and reduces metastatic dissemination in a murine lung metastasis model [194]. Based on this knowledge, a synthetic CLEC-2-blocking agent, cobalt hematoporphyrin, has been developed which inhibits CLEC-2 mediated platelet–tumour cell-interaction and curbs haematogenous metastasis in in vivo experiments [195].

### 5.3. Novel Targets for SV-CLRPs on Endothelial Cells

Rhodocetin-αβ, one of the two heterodimeric subunits of rhodocetin from *Calloselasma rhodostoma*, not only targets GPIb on platelets antagonistically, but also binds to neuropilin-1 on endothelial cells in an agonistic manner [120,151]. The molecular mechanisms of these interactions are so far unknown and not described for any other SV-CLRP. At the cellular level, rhodocetin αβ binds neuropilin-1 and forms a trimeric complex with MET, a protein tyrosine kinase receptor for hepatocyte growth factor (HGF) [196,197,198,199,200]. This elicits several pleiotropic effects in endothelial cells, among them the rearrangement of integrins and adhesomes, as well as of the adhesome-anchored actin cytoskeleton [151]. In a monolayer of coherent endothelial cells, rhodocetin-αβ induces their activation and consequently augments diapedesis of myeloid immune cells [201]. In two animal tumour models, this SV-CLRP does not primarily influence endothelial cells in normal vessels because of the inaccessibility of blood-borne rhodocetin-αβ to the basolaterally expressed neuropilin-1. Strikingly however, this results in a tumour-specific disaggregation of tumour blood vessels, in which tumour cells with their non-polarized cell expression of neuropilin-1 have integrated into the endothelial cell lining [202]. This might offer a new avenue to direct tumouricidal agents to solid tumours.

## 6. Translational Potential of SV-CLRPs into Medicine and Perspectives

The CLRP-associated snake venom proteinase, RVV-X and carinactivase-1 are diagnostically used to quantify coagulation factors [92,93,203]. Beyond this, the non-enzymatic action of SV-CLRPs to block cellular interactions with the extracellular matrix is a promising task, but so far largely at the experimental level [204]. Targeting the collagen receptor, some SV-CLRPs such as rhodocetin and sochicetin-A effectively reduce extravasation and micro-metastasis formation of tumour cells in animal cancer models [148,205].

The most advanced progress in applying SV-CLRP derived compounds in the clinics has been achieved in the field of preventing inappropriate platelet activation and aggregation, resulting in thrombotic vessel occlusion, myocardial infarct, and stroke [206,207]. In this field, the GPIb-blocking SV-CLRP agkicetin from *Agkistridon acutus* has taken the lead. Under the name “anfibatide”, it was shown to inhibit vWF-induced platelet activation and thrombus formation [108,208]. Application of anfibatide significantly reduces the infarct volume in an animal ischemic stroke model [209,210]. It has been tested for myocardial infarction in a clinical study [211]. Moreover, it might help patients suffering from thrombotic thrombocytopenia purpura, in which a deficient cleavage of multimeric vWF in blood results in enhanced thrombus formation [212,213].

Another foreseeable success of harnessing SV-CLRP in medicine will potentially be rhodocytin or a derivative of it. Not only was it key to decipher the important role of tumour cell-induced platelet activation via the podoplanin-CLEC-2 axis, but it has also been used successfully to experimentally effect haematogenous metastasis [191,194]. Most recently, a chemical compound not related to the SV-CLRP has been identified to precisely block this molecular interaction and, thus, curb metastasis of blood-borne tumour cells [195].

## 7. Conclusions

The non-enzymatic SV-CLRPs have a great potential in translational medicine because of their robust structure and their affinity towards their target molecules. However, the molecular mechanisms of their target specificities, of their inhibiting or activating functions, and of their potential to influence the corresponding cellular function agonistically or antagonistically are largely unknown. They have to be deciphered in correlation with the molecular and supramolecular structures in order to be able to fully exploit the potential of SV-CLRPs or their recombinant or synthetic derivatives in medicine.

## Abbreviations:

CLRP	C-type lectin-related protein
CTLD	C-type lectin domain
GP	glycoprotein
vWF	von Willebrand factor
